# Systematic Review of Observational Studies Assessing Bleeding Risk in Patients with Atrial Fibrillation Not Using Anticoagulants

**DOI:** 10.1371/journal.pone.0088131

**Published:** 2014-02-11

**Authors:** Luciane Cruz Lopes, Frederick A. Spencer, Ignacio Neumann, Matthew Ventresca, Shanil Ebrahim, Qi Zhou, Neera Bhatnagar, Sam Schulman, John Eikelboom, Gordon Guyatt

**Affiliations:** 1 Pharmaceutical Sciences Postgraduate Course, University of Sorocaba, Sao Paulo, Brazil; 2 Department of Medicine, Division of Cardiology, McMaster University, Hamilton, Ontario, Canada; 3 Internal Medicine Department, School of Medicine, Pontificia Universidad Catolica de Chile, Santiago, Chile; 4 Department of Clinical Epidemiology and Biostatistics, McMaster University, Hamilton, Ontario, Canada; 5 Department of Anesthesia, McMaster University, Hamilton, Ontario, Canada; 6 Stanford Prevention Research Center, Stanford University, Stanford, California, United States of America; 7 Health Sciences Library McMaster University, Hamilton, Ontario, Canada; 8 Department of Medicine, McMaster University, Hamilton, Ontario, Canada; 9 Department of Medicine, Division of Hematology and Thromboembolism, McMaster University, Hamilton, Ontario, Canada; Royal College of Surgeons, Ireland

## Abstract

**Background:**

Patients with atrial fibrillation considering use of anticoagulants must balance stroke reduction against bleeding risk. Knowledge of bleeding risk without the use of anticoagulants may help inform this decision.

**Purpose:**

To determine the rate of major bleeding reported in observational studies of atrial fibrillation patients not receiving Vitamin K antagonists (VKA).

**Data Sources:**

We searched MEDLINE, EMBASE and CINAHL to October 2011 and examined reference lists of eligible studies and related reviews.

**Study Selection:**

All longitudinal cohort studies that included over 100 adult patients with atrial fibrillation not receiving VKA.

**Data Extraction:**

Teams of two reviewers independently and in duplicate adjudicated eligibility, assessed risk of bias and abstracted study characteristics and outcomes.

**Data Synthesis:**

Twenty-one eligible studies included 96,448 patients. Major bleeding rates varied widely, from 0 to 4.69 events per 100 patient-years. The pooled estimate in 13 studies with 78839 patients was 1.59 with a 99% confidence interval of 1.10 to 2.3 and median 1.42 (interquartile range 0.62–2.70). Pooled estimates for fatal bleeding and non-fatal bleeding from 4 studies that reported these outcomes were, respectively, 0.40 (0.34 to 0.46) and 1.18 (0.30 to 4.56) per 100 patient-years. In 9 randomized controlled trials (RCTs) the median rate of major bleeding in patients not receiving either anticoagulant or antiplatelet therapy was 0.6 (interquartile 0.2 to 0.90), and in 12 RCTs the median rate of major bleeding in patients receiving a single antiplatelet agent was 0.75 (interquartile 0.4 to 1.4).

**Conclusion:**

Results suggest that patients with atrial fibrillation not receiving VKA enrolled in observational studies represent a population on average at higher risk of bleeding.

## Introduction

Atrial fibrillation is common, and incurs a major burden of morbidity and mortality largely as a result of associated stroke and systemic embolism. Anticoagulants reduce the risk of stroke or systemic embolism, but at a cost of inconvenience and an increased risk of serious bleeding. Choosing whether or not to use anticoagulants to reduce the risk of thromboembolism requires trading off an absolute reduction in stroke against an absolute increase in serious bleeding. Estimating the magnitude of the increased risk of bleeding using VKA is crucial in making decisions regarding anticoagulant use.

In a prior systematic review of the available observational studies, we have demonstrated that although major bleeding rates in atrial fibrillation patients receiving VKAs varied widely from study to study, the median major bleeding rate was 2.05 per 100 patient-years, interquartile range 1.57 to 3.35 [1], a value very close to that of warfarin-treated arms of randomized control trials (RCTs) (median 2.1, interquartile range 1.54 to 3.09). Applying the relative increase in bleeds with VKA from RCTs - 2.58 [2] - leads to an estimate of absolute increase in bleeding rate of 1.54 per 100 patient-years with warfarin use in atrial fibrillation.

Defining the bleeding risk in patients with atrial fibrillation not taking anticoagulants may provide further insight into the challenging decision regarding use of anticoagulants. We therefore undertook a systematic review and meta-analysis to define bleeding risk, including intracranial and extracranial, in representative patients in the community not receiving anticoagulants. Being aware that bleeding risk is likely to differ across patient and study characteristics, we, a priori, postulated explanations for possible heterogeneity in bleeding risk. We compared results to bleeding risks reported in the arms of randomized trials not receiving anticoagulants (either no antithrombotic therapy, placebo, or a single antiplatelet agent).

## Methods

All methodological decisions in this review were made in advance and were recorded in a prior protocol that is available on request.

### Data Sources and searches

We searched the central MEDLINE, EMBASE and CINAHL (to October 2011). We restricted the search to human subjects and adults. Medical subject headings included: hemorrhage (or bleeding$ or bleed*); atrial fibrillation (or auricular fibrillation) and risk (risk factors or risk assessment or risk*). For every eligible study, we identified, and for studies such as review articles that we identified that included citations to potentially eligible studies, one reviewer examined the reference list.

Teams of two investigators independently screened each title and abstract from this search. If either of the two screeners identified a citation as potentially relevant, we obtained the full text article for detailed review. Teams of two reviewers independently determined the eligibility of all studies that underwent full text evaluation. Disagreements were resolved through discussion between the two reviewers; when this did not resolve differences, a third reviewer made a final decision on the study's eligibility.

### Study Eligibility

We included all longitudinal cohort studies or case series that included adults with atrial fibrillation. Articles met the following criteria: a) >80% of patients enrolled have atrial fibrillation and are 18 or older or b) <80% of patients included had atrial fibrillation, but results presented for the atrial fibrillation subset separately; receiving or not receiving antiplatelet agents but not receiving VKA; ≥100 patients with atrial fibrillation not using VKA; some estimate of major bleeding in an identifiable group not receiving VKA. We excluded RCTs; studies dealing only with hospitalized patients or emergency department patients; and studies with clearly unrepresentative populations with a different bleeding risk than a representative population (e.g. prior stroke, ablation).

### Data abstraction and quality assessment

We abstracted the following information from each eligible study: period of data collection; country; source of funding; duration of follow-up taking or not taking antiplatelet agents; total number of atrial fibrillation patients not taking VKA; funding of care; age. We recorded rates of major bleeding, both fatal bleeding and non-fatal which we characterized as intracranial or extra-cranial and, for extra-cranial bleeding, further characterized as gastrointestinal or other. For all major bleeding outcomes we recorded the number of patients who had a bleed, rate of bleeding (typically number of bleeds per 100 patient years) and total number of bleeds.

We assessed risk of bias using the following criteria i. patient selection random or consecutive versus other; ii. whether investigators excluded patient groups; iii. proportion lost to follow up; iv. explicitness of bleeding criteria; v. primary data collection (as opposed to administrative data base) for bleeding from patient report, physician report, or hospital records. If reported, we noted whether patients received antiplatelet agents from a prescription or over the counter.

### Data synthesis and statistical analysis

We anticipated that bleeding rates would differ across studies and generated a priori hypotheses of what study features might be responsible for the differences: i. age distribution (older age, higher bleeding incidence); ii. gender distribution (women higher incidence); iii. ethnic groups (geographic area); iv. proportion nursing facility residence; v. proportion within each CHADS category (includes congestive heart failure, hypertension, age>75, diabetes = 1 point, history of stroke = 2 points); vi. proportion taking one antiplatelet agent; vii. proportion with current or remote bleeding event; alcohol/ drug abuse; viii. funding of health care (gradient of bleeding incidence if unadjusted by age Medicare, Medicaid, mixed and private payment); ix. risk of bias, in particular use of administrative data bases (claims data) versus primary data collection (primary data collection, higher bleeding incidence).

In keeping with standards for avoiding over fitting, we examined all hypotheses for which information was documented in at least 10 studies for the continuous independent (predictor) variables or at least 5 studies for each level of the independent categorical variables.

Our pooled analyses estimated the rate of bleeding per 100 patient-years of warfarin exposure for overall major bleeding and its subcategories. We used variance estimates based on confidence intervals, where provided, or the total number of patient-years of exposure. If rates, but neither confidence intervals nor total exposure, were available, we estimated total exposure from the mean years of follow-up. When total number of bleeds or total number of patients who bled, but not rates were available, we estimated rates based on the total number of bleeds or the proportion that bled and the mean follow-up. For instances in which one of a group of studies reported no events on a group, we used the 0.5 continuity correction for the calculation. We pooled estimates in log-scale units across studies using DerSimonian and Laird's random effects model weighted by the inverse of the variance and then back transformed to the rate in natural units. The pooled estimates were tested by Z-statistics and the heterogeneity, measured by Q-statistic, among the studies examined by the Chi-square test. When the heterogeneity was presented, a component of variance due to inter-study variation, D, was incorporated in the confidence interval calculation for the estimate. Studies that did not include any of the data above were not included in the pooled estimate; for such studies, we summarized bleeding rates descriptively. To examine the possibility of publication bias we constructed a funnel plot.

We made calculations for patients using or not using antiplatelet agents when studies reported these data. To explain heterogeneity, we performed univariable meta-regression analysis weighted by inverse of variance. The dependent variable was the logarithm of the bleeding rate in 100 patient-years; the independent variables are described above in our 9 a priori hypotheses regarding heterogeneity. For the purpose of interpretation, we back transformed the exponential function parameter to natural units and considered a threshold p-value of 0.01 to be significant.

### Bleeding rates from randomized trials

Using the atrial fibrillation article from the 9th iteration of the American College of Chest Physicians (ACCP) antithrombotic guidelines [2], we identified randomized trials comparing anticoagulants to placebo, no antithrombotic agent, or a single antiplatelet agent for stroke prevention in atrial fibrillation and abstracted the overall rates of major bleeding among subjects not taking warfarin.

## Results

**Figure 1:**
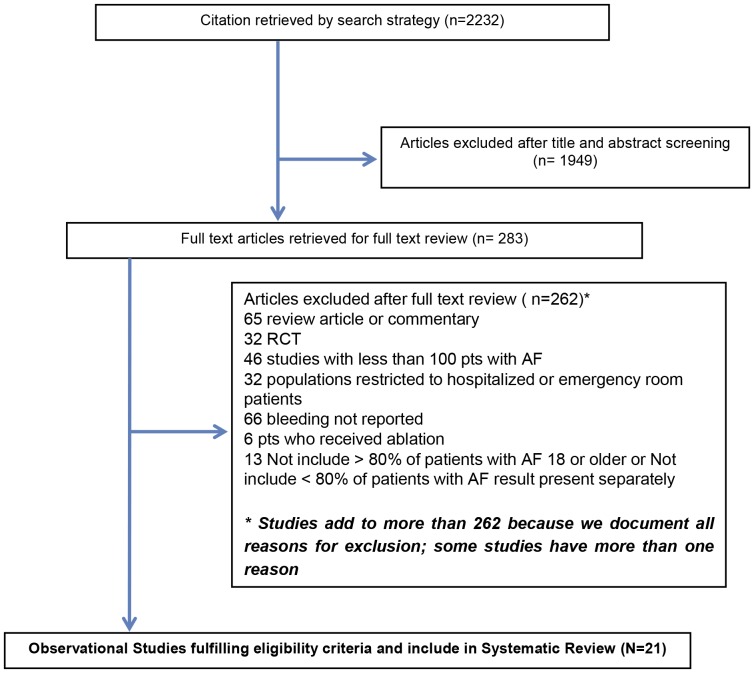
Literature search and study selection.

Our search strategy identified 2,232 citations. Of these, 283 proved potentially eligible, and 21 fulfilled eligibility criteria and are included in this systematic review (**Figure 1**).

### Study and Patient Characteristics

The 21 eligible studies, most conducted in North America and Europe, followed their patients from 1.0 to 6.0 years (mean or median), reported on between 130 and 65,477patients with a mean age varying between 63.5 and 81 years (median 67.0, interquartile range 63.5 to 76.7) of whom the majority were male, and taking at least one antiplatelet agent (Table 1, Table 2).

**Table 1:** Study characteristics of atrial fibrillation patients not taking VKA.

CHARACTERISTICS	STUDIES (N)	
**SAMPLE SIZE**		
Total number of AF patients	21	96,448
Total numbers of AF patients-years	9*	33,299.24
**GENDER**		**MEDIAN (INTERQUARTILE RANGE)**
Proportion female	11	48.6 (36.5–55.4)
**AGE**		**MEDIAN (INTERQUARTILE RANGE)**
	9	76.3 (69.3–79.1)
**USE OF ANTIPLATELET AGENTS** #		**NUMBER PTS**
Pts not taking any antiplatelet agent	9	2,602
Pts taking at least one antiplatelet agent	13	58,151
Pts taking two or more antiplatelet agent	1	2,859
Pts taking antiplatelet agents number unspecified	7	35,695
**FOLLOW UP**		**MEDIAN (INTERQUATILE RANGE)**
Minimum, days	3	180 (90–180)
Maximum, days	11	1,260 (720–1825)
Mean, days	6	655.5 (388.3–1095)
**DEFINITION MAJOR BLEEDING**		**NUMBER PTS** @
Hospitalization (initiating or prolonging)	11	75,941
Hemoglobin decrease ≥2 g/L	3	1,772
Transfusion ≥2 units blood	8	3,503
Critical area (e.g. intracranial, spinal, retroperitoneal, pericardial, hemarthrosis)	8	10,906
Surgery (requiring)	3	1,301
ICD-9 codes	10	92,230
Fatal	9	56,775
Others	5	9,313
**PUBLICATIONS YEAR**		**NUMBER PTS**
1998–2005	6	13,174
2006–2010	15	83,274
**COUNTRY**		**NUMBER PTS**
Canada	2	1,022
USA	9	34,091
Europe	7	60,616
Asia	2	422
Australia and New Zealand	1	297
**SOURCE OF FUNDING**		**NUMBER PTS**
For profit	5	69,600
Not for profit	10	11,268
Both	2	1,797
Not specified	4	13,783

@Some studies used more than one definition and all definitions are included.

* 12 studies didn't provided the number pts-year follow up.

#some studies have more than one arm.

**Table 2:** Characteristics of population included in observational studies included.

AUTHOR	MEAN FOLLOW UP (year)	TOTAL NUMBER OF ATRIAL FIBRILLATION PATIENTS NOT TAKING VKA	FEMALE (%)	AGE (mean)	OUTCOME MEASURED	MAJOR BLEEDING (PER 100 PTS-YEAR)		
						All patients not taking VKA	Patients taking antiplatelet agent	Patients not taking antiplatelet
SPAFIII, 1998 [8]	2	892	22	67	Major bleeding, intracranial, extracranial, extacranial gastrointestinal, extracanial others	0.44	0.44	NA
Jackson, 2001 [15]	3.14	297	39.6	77	Major bleeding	3.9	3.42	4.73
Leung, 2003 [6]	2	143	NA	NA	Major bleeding, Intracranial, extracranial, extacranial gastrointestinal	1.40	1.72	0
Sam, 2004 [19]	NA	313	50.1	NA	Major bleeding, intracranial,	NA	NA	NA
Currie, 2005 [16]	NA	3885	55.4	65.3*	Major bleeding	NA	NA	NA
Darkow, 2005 [13]	NA	7644	54.5	79.8	Major bleeding, intracraneal	3.1	NA	NA
Boulanger,2006 [10]	3.4	1787	65.1	76	Major bleeding,	0.26	NA	NA
Burton, 2006 [12]	3.1	372	NA	65*	Major bleeding, intracranial, extracranial, extacranial gastrointestinal	1.67	1.66	1.31
Gage, 2006 [17]	NA	2187	NA	81	Major bleeding	NA	NA	NA
Parkash, 2007 [7]	2.1	130	40	69.2	Major bleeding	2.2	NA	NA
Shen, 2007 [20]	4.9	7851	NA	41.9*	Intracranial	NA	NA	NA
Meiltz, 2008 [3]	1.01	213	69.8	NA	Major bleeding, intracranial, extracraneal, extracraneal, others	0	0	0
Wess, 2008 [22]	NA	5622	75.1	76.7	Intracranial, extracranial, extacranial gastrointestinal	NA	NA	NA
Boccuzzi, 2009 [18]	NA	2167	NA	NA	Major Bleeding	NA	NA	NA
Lai, 2009 [9]	1.9	167	36.5	77	Major bleeding, intracranial, extracranial, extacranial gastrointestinal, extracanial others	4.69	NA	NA
Singer, 2009 [21]	6	6353	48.6	48.6*	Intracranial	NA	NA	NA
Friberg, 2010 [23]	NA	617	NA	NA	Intracranial	NA	NA	NA
Hansen, 2010 [14]	NA	54,117	NA	NA	Major bleeding, intracranial, extracranial	2.7	3.84***	2.10
Lee, 2010 [4]	1.7	279	34.1	63.5	Major bleeding, intracranial, extracranial, extacranial gastrointestinal, extracanial others	0.62	0.34	1.05
Ortiz, 2010 [5]	1.91	232	NA	NA	Major bleeding	0.9	NA	NA
Pisters, 2010 [11]	1	1180	NA	49.7**	Major bleeding	1.42	0.97	NA

NA – not avaliable.

* it represents proportion of pts with threshold age ≥75 yo;

** it represents proportion of pts with threshold age ≥65 yo;

*** taking two antiplatelet agents.

### Risk of Bias

Most of studies enrolled consecutive or random patients, provided explicit criteria for bleeding and undertook some primary data collection. No study met all 6 risk of bias criteria; 15 studies met 3 or fewer criteria (Table 3, Table S1)

**Table 3:** Risk of bias.

STUDY	CONSECUTIVE OR RANDOM	NO EXCLUSIONS	SPECIFIED LOST TO FOLLOW UP	EXPLICIT CRITERIA FOR THE BLEED	DOCUMENTATION OF ANTIPLATELET USE	PRIMARY DATA COLLECTION	CRITERIA MET
SPAFIII, 1998 [8]	**✓**	**<$>\raster="rg1"<$>**	**✓**	**✓**	**✓**	**✓**	**5**
Jackson, 2001 [15]	**✓**	**<$>\raster="rg1"<$>**	**<$>\raster="rg1"<$>**	**✓**	**<$>\raster="rg1"<$>**	**✓**	**3**
Leung, 2003 [6]	**✓**	**✓**	**<$>\raster="rg1"<$>**	**✓**	**<$>\raster="rg1"<$>**	**✓**	**4**
Sam, 2004 [19]	**✓**	**✓**	**<$>\raster="rg1"<$>**	**✓**	**<$>\raster="rg1"<$>**	**✓**	**4**
Currie, 2005 [16]	**✓**	**✓**	**<$>\raster="rg1"<$>**	**<$>\raster="rg1"<$>**	**<$>\raster="rg1"<$>**	**✓**	**3**
Darkow, 2005 [13]	**✓**	**<$>\raster="rg1"<$>**	**<$>\raster="rg1"<$>**	**✓**	**<$>\raster="rg1"<$>**	**<$>\raster="rg1"<$>**	**2**
Boulanger,2006 [10]	**✓**	**<$>\raster="rg1"<$>**	**<$>\raster="rg1"<$>**	**<$>\raster="rg1"<$>**	**✓**	**<$>\raster="rg1"<$>**	**2**
Burton, 2006 [12]	**<$>\raster="rg1"<$>**	**<$>\raster="rg1"<$>**	**<$>\raster="rg1"<$>**	**✓**	**<$>\raster="rg1"<$>**	**✓**	**2**
Gage, 2006 [17]	**✓**	**<$>\raster="rg1"<$>**	**<$>\raster="rg1"<$>**	**<$>\raster="rg1"<$>**	**✓**	**<$>\raster="rg1"<$>**	**2**
Parkash, 2007 [7]	**<$>\raster="rg1"<$>**	**<$>\raster="rg1"<$>**	**<$>\raster="rg1"<$>**	**✓**	**✓**	**✓**	**3**
Shen, 2007 [20]	**✓**	**<$>\raster="rg1"<$>**	**<$>\raster="rg1"<$>**	**✓**	**<$>\raster="rg1"<$>**	**✓**	**3**
Meiltz, 2008 [3]	**✓**	**✓**	**<$>\raster="rg1"<$>**	**<$>\raster="rg1"<$>**	**<$>\raster="rg1"<$>**	**✓**	**3**
Wess, 2008 [22]	**✓**	**✓**	**<$>\raster="rg1"<$>**	**<$>\raster="rg1"<$>**	**✓**	**<$>\raster="rg1"<$>**	**3**
Boccuzzi, 2009 [18]	**✓**	**✓**	**<$>\raster="rg1"<$>**	**✓**	**<$>\raster="rg1"<$>**	**<$>\raster="rg1"<$>**	**3**
Lai, 2009 [9]	**✓**	**<$>\raster="rg1"<$>**	**<$>\raster="rg1"<$>**	**✓**	**<$>\raster="rg1"<$>**	**✓**	**3**
Singer, 2009 [21]	**✓**	**✓**	**<$>\raster="rg1"<$>**	**✓**	**<$>\raster="rg1"<$>**	**✓**	**4**
Friberg, 2010 [23]	**✓**	**<$>\raster="rg1"<$>**	**<$>\raster="rg1"<$>**	**✓**	**<$>\raster="rg1"<$>**	**✓**	**3**
Hansen, 2010 [14]	**✓**	**✓**	**<$>\raster="rg1"<$>**	**✓**	**<$>\raster="rg1"<$>**	**<$>\raster="rg1"<$>**	**3**
Lee, 2010 [4]	**✓**	**<$>\raster="rg1"<$>**	**<$>\raster="rg1"<$>**	**✓**	**✓**	**<$>\raster="rg1"<$>**	**3**
Pisters, 2010 [11]	**✓**	**<$>\raster="rg1"<$>**	**✓**	**✓**	**✓**	**✓**	**5**

### Bleeding outcomes in observational studies

**Figure 2:**
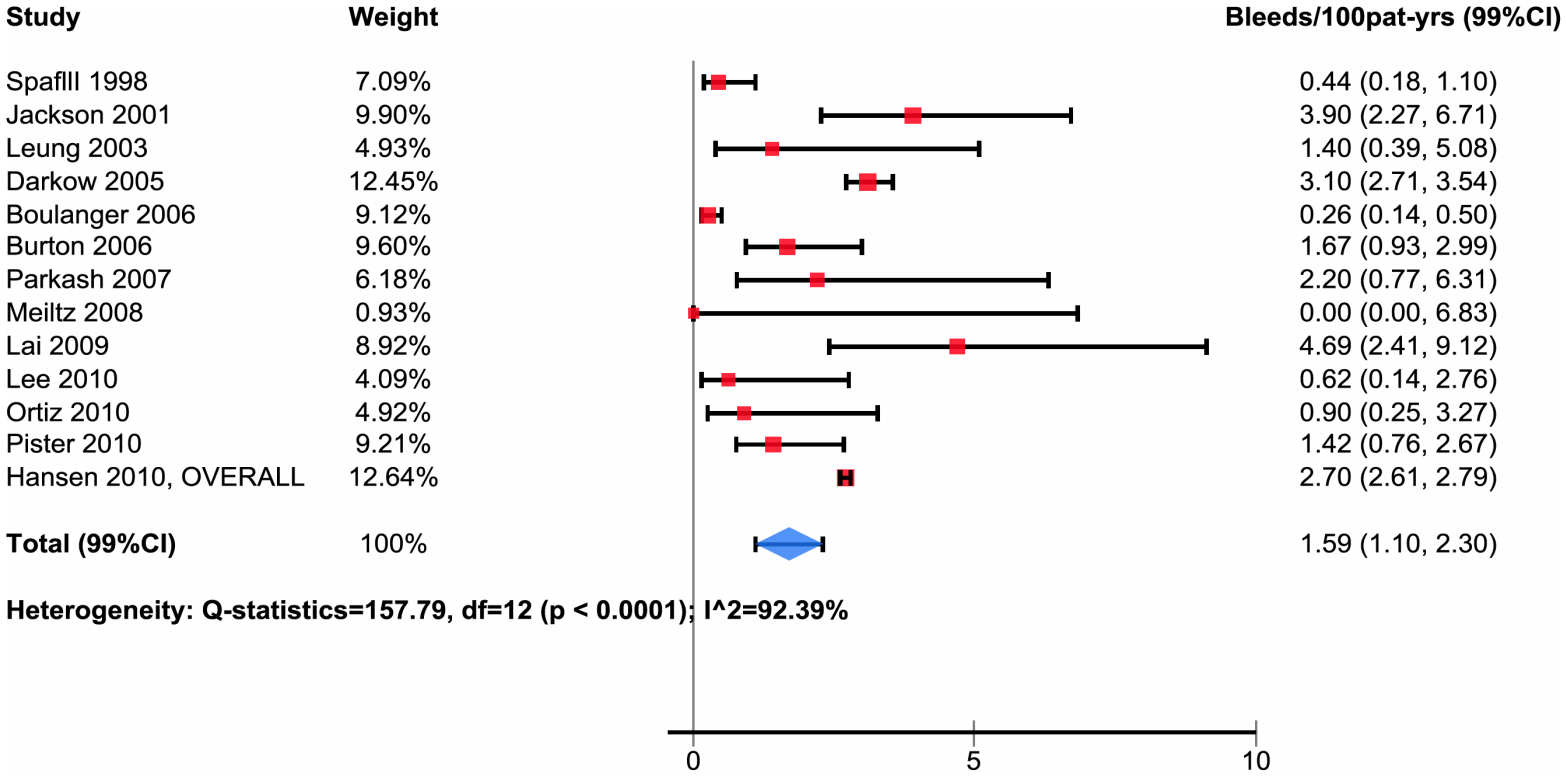
Major bleeding overall population not taking Vitamin K atagonist.

Of the 21 eligible studies, 13 reported data regarding overall major bleeding that allowed pooling as bleeds per 100 patient-years [3]–[15]. The total bleeding rates varied widely across studies - more than an order of magnitude - from 0.0 to 4.69 per 100 patient-years (median 1.42, interquartile range 0.62–2.70). Of the 13 studies, 5 reported bleeding rates of less than 1.0 per 100 patient-year, 5 between 1.0 to 2.7, and 3 rates greater than 3 per 100 patient years (Table 2, Table S2), resulting in a pooled estimate of bleeds per 100 patient-years 1.59 with 99% confidence interval 1.10 to 2.30 (Table 4, Figure 2).

**Table 4:** Rates of bleeding.

BLEEDING OUTCOMES	NUMBER STUDIES (PTS)	POOLED ESTIMATE BLEEDING RATES PER 100 PATIENT-YEARS (99% CI) or range
**MAJOR BLEEDING (pooled)**		
**Total, n = 13**	78839	1.59 (1.10, 2.30), 0–4.69
**Fatal, n = 4**	48405	0.40 (0.34, 0.46), 0.18–0.62
**Non fatal, n = 4**	48405	1.18 (0.30, 4.56), 0.09–3.30
**MAJOR BLEEDING IN NON-POOLABLE STUDIES (RANGE)**		
**INTRACRANIAL BLEEDING (pooled)**		
**Total, n = 8**	64072	0.32 (0.17, 0.59), 0.06–0.94
**Fatal, n = 2**	651	0.35 (0.07, 1.84), 0.17–0.62
**Non fatal, n = 2**	651	0.15 (0.03, 0.77), 0.09–0.17
**EXTACRANIAL (pooled)**		
**Total, n = 3**	1338	0.68 (0.05, 9.48), 0.09–3.74
**Fatal, n = 2**	651	0.09 (0.01, 0.72), 0.08–0.09
**Non fatal, n = 1**	279	0.62 (0.14, 2.76)
**GATROINTESTINAL (pooled)**		
**Total, n = 4**	48879	0.89 (0.29, 2.69), 0.09–1.87
**Fatal, n = 2**	651	0.09 (0.01, 0.72), 0.08–0.09
**Non fatal, n = 1**	279	0.62 (0.14, 2.76)
**OTHERS (pooled)**		
**Total, n = 3**	1338	0.25 (0.01, 8.34), 0.06–1.87
**Fatal, n = 1**	279	0.62 (0.14, 2.76)
**Non fatal, n = 1**	279	0.62 (0.14, 2.76)

**Figure 3:**
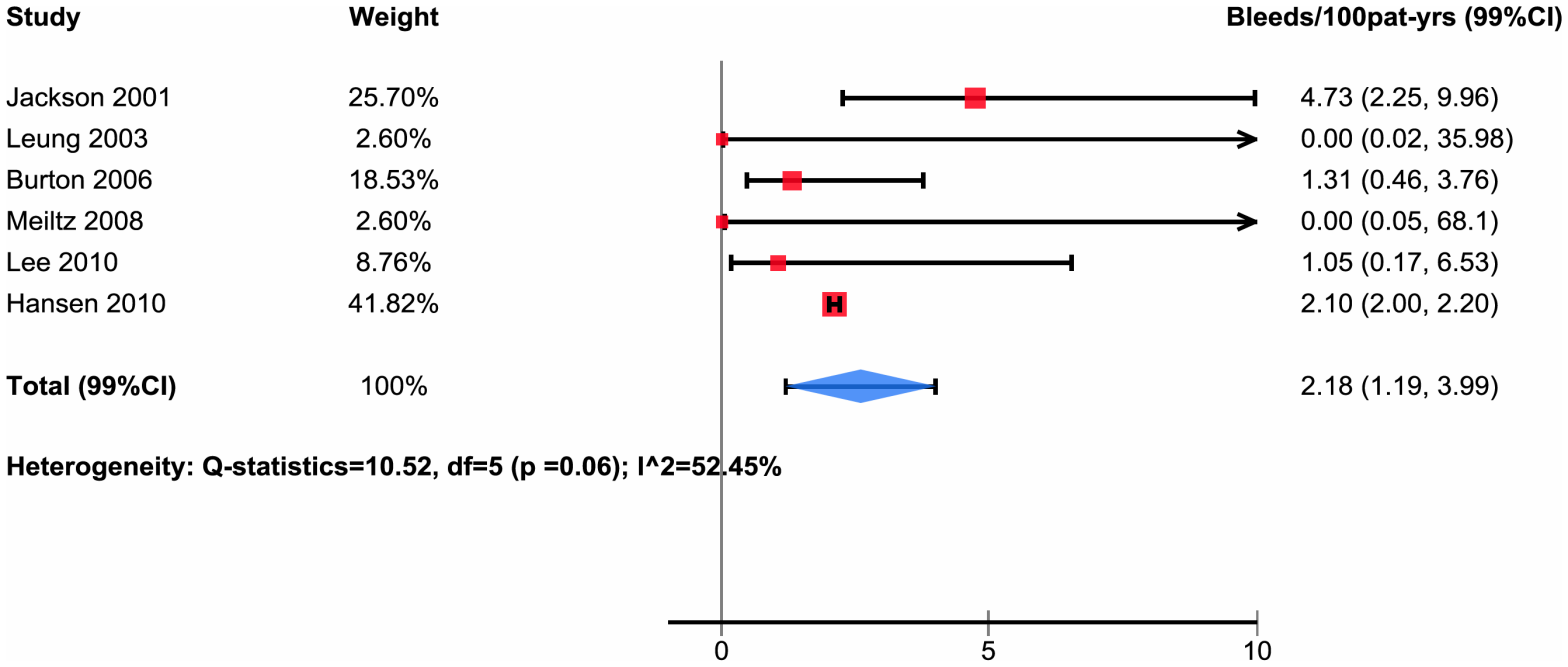
Subpopulation not taking antiplatelet.

Of 21 studies, 8 reported data major bleeding in patients taking antiplatelet agents with rates from 0.0 to 3.84 per 100 patient-years (median 1.32, interquartile range 0.39 to 2.57) and pooled estimate of bleeds per 100 patient-years 1.35 with 99% confidence interval 0.55 to 3.28 (Table 2, Figure 3).

**Figure 4:**
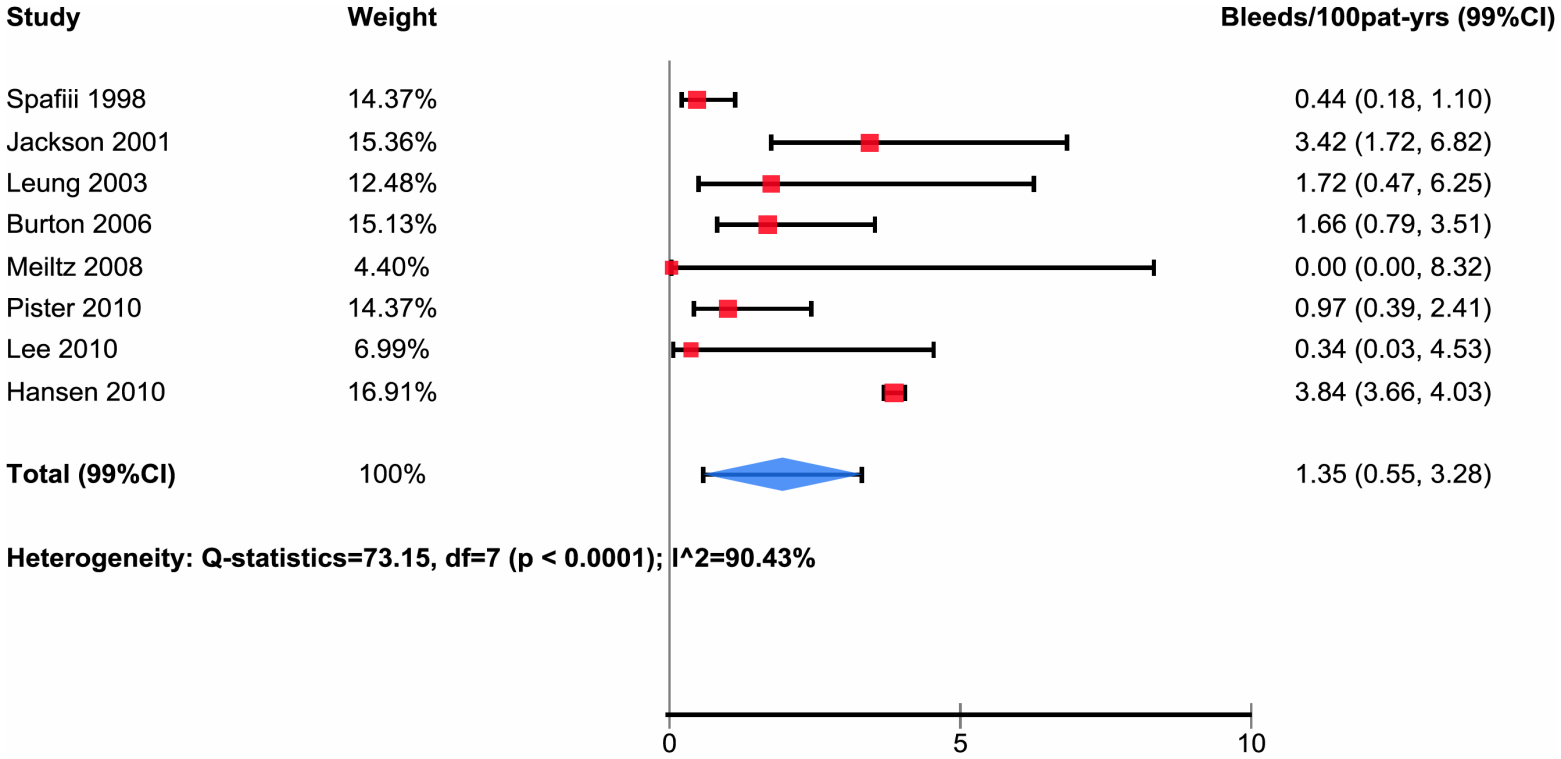
Subpopulation taking at least one antiplatelet.

Of the 21 studies, 6 reported major bleeding in patients not taking antiplatelet agents with overall bleeding rates from 0.0 to 4.73 per 100 patient-years (median 1.54, interquartile range 1.05 to 2.10) and pooled estimate of bleeds per 100 patient-years 2.18 with 99% confidence interval 1.19 to 3.99 (Table 2, Figure 4).

Of 21 studies, 4 separately fatal (pooled estimate 0.40 per 100 patient-years) and 4 reported non-fatal (1.18 per 100 patient-years) major bleeding (Table 4, Table S2).

The 8 studies that did not report overall bleeding in a manner that allowed pooling reported overall major bleeding rates of 1.54 [16], 5.1 [17] and 6.5 [18]; the others did not report overall major bleeding rates [19]–[23]. Of these 8 studies, 5 reported results for intracranial bleeding [19]–[23] and 1 for extracranial bleeding [18] (Table S2).

Table 4 summarizes pooled results for bleeding subcategories. We observed similar large variability in bleeding rates for subcategories as we did for overall bleeding. For instance, major extracranial bleeding ranged from 0.09 to 3.74 major bleeds per 100 patient-years.

The funnel plots did not suggest publication bias (Figure S1).

### Possible determinants of variability in bleeding rates

Sufficient data were available to explore only 2 categories of variables to determine whether they were predictors of major bleeding events: use of administrative data bases (claims data) versus primary data collection, and average age of patients.

With respect to data collection, studies fell into three categories: exclusively primary data collection: major bleeding rate per 100 patient-years (99%CI) 2.30 (0.27, 19.40); exclusively administrative data base data collection: 2.71 (2.20, 3.33); and both sources of data collection: 0.28 (0.04, 1.75). Bleeding rates were significantly higher with exclusively administrative data collection versus both data sources (p = 0.003) and of borderline significance in primary data collection versus both data sources (p = 0.04). There were only 2 studies with both primary and administrative data base data collection and one of these, with an extremely low bleeding rate of 0.15 per 100 patient-years, drove this result.

We were unable to demonstrate a statistical association with age and bleeding rates, estimate of relative change (relative risk for each increase in mean age of one year 1.02, 95% CI 0.94 to 1.11, p = 0.11).

### Bleeding rates in randomized trials

The 9th iteration of the ACCP Antithrombotic guidelines [2] summarized 9 RCTs that included an arm in which patients received no antithrombotic prophylaxis (total sample size of 4,870) and 12 RCTs that included an arm in which patients received one antiplatelet agent (total sample size of 5,734). The mean age in these cohorts varied from 63.3 to 81.1 years (interquartile range 67 to 73 years) with the same preponderance of males seen in the observational studies (median proportion of females 38.2%, interquartile range 30.1 to 44.2) (Table 4). The median rate of major bleeding in patients not receiving treatment was 0.6, (interquartile range 0.20 to 0.90, total range 0.0 to 1.9 bleeds per 100 patient-years) and in the group receiving one antiplatelet was 0.75, (interquartile range 0.24 to 1.30, total range 0.0 to 2.0 bleeds per 100 patient-years).

## Discussion

We identified 21 observational studies of patients with atrial fibrillation not receiving VKA of which 13 reported data contributing to pooled estimates of total major bleeding rate of 1.59 per 100 patient-years with 99% confidence interval of 1.10 to 2.3 and median 1.42 (interquartile range 0.62 to 2.70) (Table 4, Figure 2). Patients in the NVKA arms of RCTs of alternative management strategies for patients with atrial fibrillation experienced appreciably lower bleeding rates (Table 5).

**Table 5:** Characteristics of RCTs including atrial fibrillation patients.

STUDY	PATIENTS (N)	FEMALE (%)	AGE (mean)	Major bleeding (per 100 pt year)	
				No antiplatelet	Taking at least one antiplatelet agent
**AFASAK, 1989 ** [**24**]	336	53.7	72.6	0.0	
**BAATAF, 1990 ** [**25**]	213	25.0	68.5	0.2	
**JAST, 2006 ** [**26**]	445	30.3	64.8	0.2	
**Edvardsson, 2003 ** [**27**]	334	39	73.0	0.5	
**EAFT, 1993 ** [**28**] **,**	378	47.0	73.0	0.6	
**CAFA, 1991 ** [**29**]	191	26.7	67.4	0.8	
**SPINAF, 1992 ** [**30**]	265	44.0	67.0	0.9	
**AFI, 1994 ** [**31**]	2140	31.5	69.5	1.0	
**SPAF, 1991 ** [**32**]	568	30	67.0	1.9	
**Hu et al., 2006 ** [**33**]	335	40.3	63.3		0.0
**SIFA, 1997 ** [**34**]	462	44.5	72.8		0.2
**AFASAK, 1989 ** [**24**]	336	53.7	72.6		0.2
**NASPEAF, 2004 ** [**35**]	242	43.0	69.9		0.35
**EAFT, 1993 ** [**28**] *	404	42.0	71.0		0.7
**EAFT, 1993 ** [**28**] *	404	42.0	71.0		0.7
**JAST, 2006 ** [**26**]	426	28.9	65.5		0.8
**SPAFII,1994 ** [**36**]	357	24.0	64.0		0.9
**AFI, 1994 ** [**31**]	888	35.0	70.0		1.0
**AFASAK 2, 1999 ** [**37**]	336	35.0	73.1		1.4
**SPAF, 1991 ** [**32**]	552	29	67.0		1.4
**PATAF, 1997 ** [**38**]	319	60.0	75.6		1.4
**SPAFII,1994 ** [**36**]	188	30.3	80.0		1.6
**BAFTA, 2007 ** [**39**]	485	46.0	81.1		2.0

* The EAFT study has two group of patients taking no VKA.

Our study fulfilled criteria for a rigorous systematic review. We specified explicit eligibility criteria; conducted a comprehensive search; assessed risk of bias using criteria specific to this review; and teams of two reviewers independently assessed eligibility, risk of bias, and checked data abstraction. Most studies met at least 3 risk of bias criteria. In particular, virtually all studies enrolled consecutive patients, specified explicit major bleeding criteria, and documented antiplatelet exposure (though most did not report bleeding rates separately for those using and not using antiplatelet agents) (Table 3, Table S1). The funnel plot for overall bleeding did not suggest publication bias (Figure S1). We conducted appropriate data analyses examining the rate of bleeding per 100 patient-years.

Limitations of our review include variability in definitions of major bleeding across studies and variability in results across studies. With only 13 studies reporting overall bleeding, some of which did not report the explanatory variables of interest, we were unable to satisfactorily explore sources of heterogeneity. The only variables we were able to formally address were the source of the data and age.

We found that studies using only administrative data and those using only primary data collection showed higher bleeding rates than studies that used both sources of information. This finding is almost certainly due to chance. The result does not conform to our a priori hypotheses, there were only two studies with both sources of data, and the result is driven almost exclusively by one of these studies. Thus, we conclude this subgroup hypothesis has low credibility.

We found no relation between mean age and bleeding risk (relative risk for each increase in mean age of one year 1.02, 95% CI 0.94 to 1.11, p = 0.11). Studies that separately reported bleeding rates in patients taking antiplately agents versus those not taking antiplatelet agents paradoxically suggested higher bleeding rates in those not taking antiplatelet agents (Figures 3 and 4).

Few studies provided information about bleeding subcategories, and those that did showed highly variable results. Once again, the paucity of studies and limited reporting precluded a systematic exploration of sources of heterogeneity.

We found no prior systematic review of bleeding rates in atrial fibrillation patients not receiving anticoagulants. Thus, our review adds original information not previously available.

Our finding that bleeding rates were appreciably higher in observational studies of atrial fibrillation patients not receiving VKAs (whether or not they received antiplatelet agents) than in the corresponding RCTs suggests that the RCTs enrolled patients at lower risk. The question then arises: which set of data is more appropriate for helping estimate bleeding risk in atrial fibrillation patients under consideration for use of anticoagulant prophylaxis?

Were we to apply the relative effect of VKAs from the RCTs (2.58) to our pooled estimate of risk of bleeding from the current observational studies (1.59) we would obtain a bleeding risk with VKAs (3.90) appreciably greater than we observed either in observational studies of patients receiving VKA or in the VKA arms of randomized trials. This strongly suggests that the cohorts enrolled in the current review included atrial fibrillation patients whose high risk of bleeding may explain their not receiving anticoagulation - that is, clinicians taking care of these patients were reluctant to prescribe anticoagulation in the face of a perceived (and it turns out accurately perceived) increased risk of bleeding.

Our results therefore highlight the limitations of observational studies in which the reasons patients did or did not receive an intervention are not completely - or not at all - transparent (sometimes referred to as confounding by indication). This lack of information is particularly limiting in the inferences that the evidence provides because of the very high variability in bleeding rates across the studies.

The bleeding incidence in patients receiving VKA from our prior review of observational studies (2.51, per 100 patient-years; median 2.05, interquartile range 1.57 to 3.35) [1] remains the best estimate of bleeding risk in patients not receiving VKA. Our prior estimate of the increase in bleeding risk with anticoagulants (1.42 per 100 patient-years) remains the best estimate of the increase in risk attendant on VKA use.

## Supporting Information

Checklist S1 (DOC)

Figure S1 Funnel Plot.
(TIF)

Table S1 Summarized Risk of bias.
(DOCX)

Table S2 Overall and subcategory bleeding rates.
(DOCX)
