# Supplementary figures and images for: Systematic Review of Observational Studies Assessing Bleeding Risk in Patients with Atrial Fibrillation Not Using Anticoagulants
Source: PLoS One. 2014 Feb 11;9(2):e88131. doi: 10.1371/journal.pone.0088131 (PMC3921139; doi:10.1371/journal.pone.0088131)

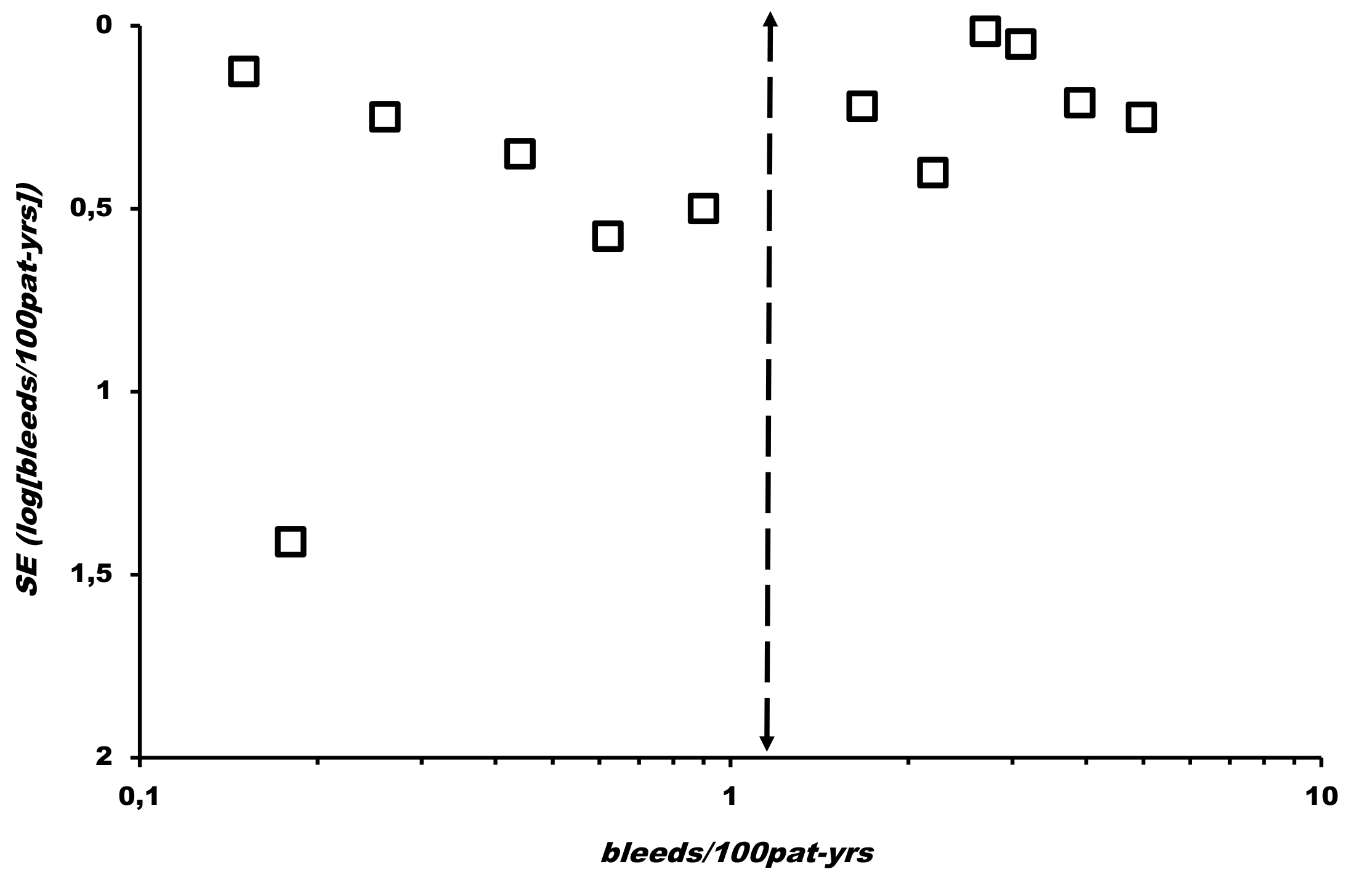

Supplement: Figure S1 — Funnel Plot. (TIF) [file pone.0088131.s002.tif]
