# Supplementary material for: Systematic Review of Observational Studies Assessing Bleeding Risk in Patients with Atrial Fibrillation Not Using Anticoagulants
Source: PLoS One. 2014 Feb 11;9(2):e88131. doi: 10.1371/journal.pone.0088131 (PMC3921139; doi:10.1371/journal.pone.0088131)
Supplement: Table S1 — Summarized Risk of bias. (DOCX) [file pone.0088131.s003.docx]

**SUPPORTING INFORMATION - APPENDIX**

**Table S1. Summarized Risk of bias**

| **CRITERIA** | **Study (n)** |
| --- | --- |
| **CONSECUTIVE OR RANDOM PTS** | |
| Yes | 18 |
| Probably yes | 1 |
| Probably no | 0 |
| No | 2 |
| **SPECIFIED THAT NO PATIENT GROUPS EXCLUDED** | |
| Yes | 9 |
| Probably yes | 0 |
| Probably no | 0 |
| No | 12 |
| **EXPLICIT CRITERIA FOR THE BLEED** | |
| Yes | 16 |
| Probably yes | 0 |
| Probably no | 0 |
| No | 5 |
| **LOST TO FOLLOW UP** |  |
| < 10% lost to follow-up | 2 |
| 10% or more lost or not reported | 19 |
| **DOCUMENTATION OF ANTIPLATELET EXPOSURE** | |
| Prescription and OTC documented | 3 |
| Prescription documented; OTC not clear | 10 |
| Prescription documented not OTC | 0 |
| No documentation of antiplatelet use | 6 |
| Unclear | 2 |
| **DATA SOURCES** |  |
| Some primary data collectio**n** | 15 |
| Administrative data only | 6 |
| **CRITERIA MET** | |
| 6 criteria | 0 |
| 5 criteria | 2 |
| 4 criteria | 4 |
| 3 criteria | 11 |
| 2 criteria | 4 |
|  |  |
