# Supplementary material for: Systematic Review of Observational Studies Assessing Bleeding Risk in Patients with Atrial Fibrillation Not Using Anticoagulants
Source: PLoS One. 2014 Feb 11;9(2):e88131. doi: 10.1371/journal.pone.0088131 (PMC3921139; doi:10.1371/journal.pone.0088131)
Supplement: Table S2 — Overall and subcategory bleeding rates. (DOCX) [file pone.0088131.s004.docx]

**Table S2. Overall and subcategory bleeding rates**

| Study | MAJOR BLEEDING | | | INTRACRANIAL | | | EXTRACRANIAL | | | EXTRACRANIAL-GASTROINTESTINAL | | | EXTRACRANIAL- OTHERS | | |
| --- | --- | --- | --- | --- | --- | --- | --- | --- | --- | --- | --- | --- | --- | --- | --- |
|  | **TOTAL** | **FATAL** | **NON-FATAL** | **TOTAL** | **FATAL** | **NON-FATAL** | **TOTAL** | **FATAL** | **NON-FATAL** | **TOTAL** | **FATAL** | **NON-FATAL** | **TOTAL** | **FATAL** | **NON-FATAL** |
| SPAFIII, 1998[[1](#_ENREF_1)] | 0.44 |  |  | 0.06 |  |  | 0.39 |  |  | 0.40 |  |  | 0.06 |  |  |
| Jackson, 2001[[2](#_ENREF_2)] | 3.90 |  |  |  |  |  |  |  |  |  |  |  |  |  |  |
| Leung, 2003[[3](#_ENREF_3)] | 1.40 |  |  |  |  | 1.29 | 0.43 |  |  | 0 |  |  | 0 |  |  |
| Sam*, 2004[[4](#_ENREF_4)] |  |  |  | 2.00 |  |  |  |  |  |  |  |  |  |  |  |
| Currie*, 2005[[5](#_ENREF_5)] |  |  |  |  |  |  |  |  |  |  |  |  |  |  |  |
| Darkow, 2005[[6](#_ENREF_6)] | 3.10 |  |  |  |  |  |  |  |  |  |  |  |  |  |  |
| Boulanger,2006[[7](#_ENREF_7)] | 0.26 |  |  |  |  |  |  |  |  |  |  |  |  |  |  |
| Burton, 2006[[8](#_ENREF_8)] | 1.67 | 0.25 | 1.27 | 0.34 | 0.17 | 0.17 |  | 0.08 |  |  | 0.08 |  |  |  |  |
| Gage, 2006[[9](#_ENREF_9)] |  |  |  |  |  |  |  |  |  |  |  |  |  |  |  |
| Parkash, 2007[[10](#_ENREF_10)] | 2.20 |  |  |  |  |  |  |  |  |  |  |  |  |  |  |
| Shen, 2007[[11](#_ENREF_11)] |  |  |  | 0.15 |  |  |  |  |  |  |  |  |  |  |  |
| Meiltz, 2008[[12](#_ENREF_12)] | 0 | 0 | 0 |  |  |  |  |  |  |  |  |  |  |  |  |
| Wess*, 2008[[13](#_ENREF_13)] |  |  |  | 1.10 | 0.18 | 0.92 |  | 0.27 |  | 8.5 | 0.27 | 8.2 |  |  |  |
| Boccuzzi*, 2009[[14](#_ENREF_14)] |  |  |  |  |  |  |  |  |  |  |  |  |  |  |  |
| Lai, 2009[[15](#_ENREF_15)] | 4.69 |  |  | 0.94 |  |  | 3.75 |  |  | 1.87 |  |  | 1.87 |  |  |
| Singer, 2009[[16](#_ENREF_16)] |  |  |  | 0.32 |  |  |  |  |  |  |  |  |  |  |  |
| Friberg, 2010[[17](#_ENREF_17)] |  |  |  | 0.23 |  |  |  |  |  |  |  |  |  |  |  |
| Hansen, 2010[[18](#_ENREF_18)] | 2.70 | 0.54 | 4.77 | 0.56 |  |  |  |  |  | 2.03 |  |  |  |  |  |
| Lee, 2010[[19](#_ENREF_19)] | 0.62 |  |  |  |  |  |  |  |  |  |  |  |  |  |  |
| Ortiz, 2010[[20](#_ENREF_20)] | 0.90 |  |  |  |  |  |  |  |  |  |  |  |  |  |  |
| Pisters, 2010[[21](#_ENREF_21)] | 1.42 |  |  |  |  |  |  |  |  |  |  |  |  |  |  |

* article provided data only for proportions, not rate

Reference

1. SPAF (1998) Patients with nonvalvular atrial fibrillation at low risk of stroke during treatment with aspirin: Stroke Prevention in Atrial Fibrillation III Study. The SPAF III Writing Committee for the Stroke Prevention in Atrial Fibrillation Investigators. JAMA 279: 1273-1277.

2. Jackson SL, Peterson GM, Vial JH, Daud R, Ang SY (2001) Outcomes in the management of atrial fibrillation: clinical trial results can apply in practice. Internal Medicine Journal 31: 329-336.

3. Leung CS, Tam KM (2003) Antithrombotic treatment of atrial fibrillation in a regional hospital in Hong Kong. Hong Kong Medical Journal 9: 179-185.

4. Sam C, Massaro JM, D'Agostino RB, Sr., Levy D, Lambert JW, et al. (2004) Warfarin and aspirin use and the predictors of major bleeding complications in atrial fibrillation (the Framingham Heart Study). American Journal of Cardiology 94: 947-951.

5. Currie CJ, Jones M, Goodfellow J, McEwan P, Morgan CL, et al. (2006) Evaluation of survival and ischaemic and thromboembolic event rates in patients with non-valvar atrial fibrillation in the general population when treated and untreated with warfarin. Heart 92: 196-200.

6. Darkow T, Vanderplas AM, Lew KH, Kim J, Hauch O (2005) Treatment patterns and real-world effectiveness of warfarin in nonvalvular atrial fibrillation within a managed care system. Current Medical Research & Opinion 21: 1583-1594.

7. Boulanger L, Hauch O, Friedman M, Foster T, Dixon D, et al. (2006) Warfarin exposure and the risk of thromboembolic and major bleeding events among medicaid patients with atrial fibrillation. Annals of Pharmacotherapy 40: 1024-1029.

8. Burton C, Isles C, Norrie J, Hanson R, Grubb E (2006) The safety and adequacy of antithrombotic therapy for atrial fibrillation: a regional cohort study. British Journal of General Practice 56: 697-702.

9. Gage BF, Yan Y, Milligan PE, Waterman AD, Culverhouse R, et al. (2006) Clinical classification schemes for predicting hemorrhage: results from the National Registry of Atrial Fibrillation (NRAF). American Heart Journal 151: 713-719.

10. Parkash R, Wee V, Gardner MJ, Cox JL, Thompson K, et al. (2007) The impact of warfarin use on clinical outcomes in atrial fibrillation: a population-based study. Canadian Journal of Cardiology 23: 457-461.

11. Shen AY-J, Yao JF, Brar SS, Jorgensen MB, Chen W (2007) Racial/ethnic differences in the risk of intracranial hemorrhage among patients with atrial fibrillation. Journal of the American College of Cardiology 50: 309-315.

12. Meiltz A, Zimmermann M, Urban P, Bloch A, Association of Cardiologists of the Canton of G (2008) Atrial fibrillation management by practice cardiologists: a prospective survey on the adherence to guidelines in the real world. Europace 10: 674-680.

13. Wess ML, Schauer DP, Johnston JA, Moomaw CJ, Brewer DE, et al. (2008) Application of a decision support tool for anticoagulation in patients with non-valvular atrial fibrillation. Journal of General Internal Medicine 23: 411-417.

14. Boccuzzi SJ, J. M, J. S, C. K, J. F, et al. (2009) Retrospective study of total healthcare costs associated with chronic nonvalvular atrial fibrillation and the occurrence of a first transient ischemic attack, stroke or major bleed. Current Medical Research and Opinion 25: 2853-2864.

15. Lai HM, W.S. A, P. K, S. A, K. P, et al. (2009) Incidence of thromboembolic stroke and of major bleeding in patients with atrial fibrillation and chronic kidney disease treated with and without warfarin. International Journal of Nephrology and Renovascular Disease 2.

16. Singer DE, Chang Y, Fang MC, Borowsky LH, Pomernacki NK, et al. (2009) The net clinical benefit of warfarin anticoagulation in atrial fibrillation.[Summary for patients in Ann Intern Med. 2009 Sep 1;151(5):I36; PMID: 19721014]. Annals of Internal Medicine 151: 297-305.

17. Friberg L, Hammar N, Rosenqvist M (2010) Stroke in paroxysmal atrial fibrillation: report from the Stockholm Cohort of Atrial Fibrillation. European Heart Journal 31: 967-975.

18. Hansen ML, Sørensen R, Clausen MT, Fog-Petersen ML, Raunsø J, et al. (2010) Risk of bleeding with single, dual, or triple therapy with warfarin, aspirin, and clopidogrel in patients with atrial fibrillation. Archives of Internal Medicine 170: 1433-1441.

19. Lee BH, Park JS, Park JH, Park JS, Kwak JJ, et al. (2010) The effect and safety of the antithrombotic therapies in patients with atrial fibrillation and CHADS score 1. Journal of Cardiovascular Electrophysiology 21: 501-507.

20. Ortiz MR, E. R, D. M, M. D, M. A, et al. (2010) Oral anticoagulation in nonvalvular atrial fibrillation in clinical practice: Impact of CHADS2 score on outcome. Cardiology 115: 200-204.

21. Pisters R, Lane DA, Nieuwlaat R, de Vos CB, Crijns HJGM, et al. (2010) A novel user-friendly score (HAS-BLED) to assess 1-year risk of major bleeding in patients with atrial fibrillation: the Euro Heart Survey. Chest 138: 1093-1100.
